# Draft genome sequences of two *Salmonella* Uzaramo isolates from poultry in South Africa

**DOI:** 10.1128/mra.01026-23

**Published:** 2023-12-15

**Authors:** Musafiri Karama, Opeyemi U. Lawal, Valeria R. Parreira, Mitra Soni, Yanhong Chen, Beniamino T. Cenci-Goga, Luca Grispoldi, Janita Greyling, Lawrence Goodridge

**Affiliations:** 1 Veterinary Public Health Section, Department of Paraclinical Sciences, Faculty of Veterinary Science, University of Pretoria, Onderstepoort, South Africa; 2 Canadian Research Institute for Food Safety (CRIFS), Department of Food Science, University of Guelph, Guelph, Ontario, Canada; 3 Department of Veterinary Medicine, Laboratorio di Ispezione Degli Alimenti di Origine Animale, University of Perugia, Perugia, Italy; 4 Department of Veterinary Tropical Diseases, Faculty of Veterinary Science, University of Pretoria, Onderstepoort, South Africa; Rochester Institute of Technology, Rochester, New York, USA

**Keywords:** draft genomes, *Salmonella*, Uzaramo, poultry, South Africa

## Abstract

*Salmonella enterica* is a zoonotic pathogen and a leading cause of foodborne gastroenteritis in humans. Here, we report the draft genome sequences of two *Salmonella* Uzaramo isolates, which were isolated from poultry organs during routine post-mortem examination in South Africa. Currently, whole-genome sequences on *Salmonella* Uzaramo are scanty.

## ANNOUNCEMENT

*Salmonella enterica* is an important zoonotic pathogen and a leading cause of foodborne gastroenteritis in humans (1). More than 2,600 *Salmonella* serovars have been reported globally, most of which have the potential to cause disease in animals and humans (1, 2). Poultry is a common reservoir of *Salmonella* serovars (3, 4). Here, we report the draft genome sequences of two *Salmonella* Uzaramo isolates, which were isolated from poultry in 2001, at the Bacteriology Laboratory, Faculty of Veterinary Science, University of Pretoria, South Africa.

Initially, *Salmonella* was detected by adding organ samples (25 g) to 225 mL of buffered peptone water and incubated at 37°C for 16–18 hours. The buffered peptone water culture (100 µL) was inoculated into 10 mL Rappaport-Vassiliadis medium and incubated at 42°C for 24 hours. After 24 hours, the cultures were spread on XLD medium and incubated for 24 hours at 37°C. Suspect *Salmonella* colonies were primarily identified by Gram staining, catalase, oxidase, and spot indole tests. Presumptive *Salmonella* isolates were subjected to secondary identification by API10S and serotyping was by the Kauffman-White method.

Frozen *Salmonella* isolates were subcultured onto tryptic soy broth and incubated for 24 hours at 35°C. Cultures were first streaked on *Salmonella Shigella* Agar followed by Brilliance *Salmonella* Agar Base (BSA). Pure colonies from BSA were used for phenotypic identification using VITEK (Biomerieux, Canada). DNA extraction and sequencing were performed on a pure colony as described previously (5, 6). Briefly, DNA was extracted using the DNeasy Blood and Tissue Kit (Qiagen, Hilden, Germany). DNA libraries were prepared using the Illumina DNA Prep Tagmentation Kit and Integrated DNA Technologies for Illumina DNA/RNA unique dual indexes (5, 6). Paired-end (2  ×  150 bp) sequencing was performed on the Illumina MiniSeq system. Default parameters were used in all bioinformatics tools. Raw reads were preprocessed with FastQC v0.11.9 (https://github.com/s-andrews/FastQC) and Trimmomatic v0.39 (7). Reads with Phred scores above 20 were assembled *de novo* using SKESA v2.4.0 (8). The assembly quality was assessed using QUAST v5.2 (9). Genomes were annotated using the NCBI Prokaryotic Genome Annotation Pipeline v6.6 (10). Isolates were serotyped using SISTR v1.0 (11), and the sequence types were determined using the PubMLST scheme (12). Resistome was identified using the CARD database (13), while the prophageome and plasmidome were determined using PHASTER (14) and MOB-suite v3.1 (15), respectively.

Sequencing of *Salmonella* SE-143 yielded 25 contigs and genome size of 4,570,753 bp with 86× coverage and % GC content of 52.2 (Table 1), whereas *Salmonella* SE-144 produced 24 contigs, 4,677,087 bp genome size with 76× coverage and % GC of 52.2. The two *Salmonella* isolates were serotyped as *Salmonella* Uzaramo. *Salmonella* Uzaramo SE-143 contains 4,221 protein-coding genes, 68 tRNAs, 9 ncRNAs, and 2 CRISPR arrays, whereas *Salmonella* Uzaramo SE-144 has 4,334 protein-coding genes, 69 tRNAs, 10 ncRNAs, and 2 CRISPR arrays.

**TABLE 1:** Summary of sequence metrics of the two historic *Salmonella* Uzaramo isolates recovered from poultry in South Africa

ID	Collection date	Isolation source	No. of contigs	Genome size (bp)	% GC	*N*_50_ (bp)	Coverage	Plasmid type	AMR gene profile	Intact phages	Protein-coding genes	Accession
*Salmonella* Uzaramo SE-143	2001	Poultry organ	25	4,570,753	52.2	397,720	85.56×	None	*aac(6')-Iaa, mdtK, sdiA, fosA, golS, mdsABC*	2 [contig 1: 343,135–384,569; contig 10: 61,564–92,748]	4,221	JAWDKR000000000.1
*Salmonella* Uzaramo SE-144	2001	Poultry organ	24	4,677,087	52.2	506,981	75.52×	IncI-gamma/K1	*aac(6')-Iaa, mdtK, sdiA, fosA, golS, mdsABC, TEM-1, sul2*	2 [contig 4; 166,061–207,495; contig 10: 61,564–92,748]	4,334	JAWDKQ000000000.1

The two *Salmonella* Uzaramo isolates belonged to the same genetic background as determined by PubMLST, having the same allele profile that constitutes a novel sequence type, and carried two intact prophages (Table 1). In addition, the resistome content was similar between the two isolates, but *Salmonella* Uzaramo SE-144 carried two additional genes encoding resistance to sulfonamides (*sul2*) and β-lactams (*TEM-1*) (Table 1). Likewise, SE-144 carried a conjugative IncI-gamma/K1 plasmid type that was absent in SE-143.

## Data Availability

These whole-genome sequences for *Salmonella* Uzaramo SE-143 and *Salmonella* Uzaramo SE-144 have been deposited at DDBJ/ENA/GenBank under the accession numbers JAWDKR000000000.1 and JAWDKQ000000000.1 and the SRA accession numbers SRR26197577 and SRR26197576. The versions described in this paper are JAWDKR000000000.1 and JAWDKQ000000000.1.
